# Autophagy Attenuates Diabetic Glomerular Damage through Protection of Hyperglycemia-Induced Podocyte Injury

**DOI:** 10.1371/journal.pone.0060546

**Published:** 2013-04-11

**Authors:** Li Fang, Yang Zhou, Hongdi Cao, Ping Wen, Lei Jiang, Weichun He, Chunsun Dai, Junwei Yang

**Affiliations:** Center for Kidney Disease, 2^nd^ Affiliated Hospital, Nanjing Medical University, Nanjing, Jiangsu Province, China; Fondazione IRCCS Ospedale Maggiore Policlinico & Fondazione D'Amico per la Ricerca sulle Malattie Renali, Italy

## Abstract

Despite the recent attention focused on the important role of autophagy in maintaining podocyte homeostasis, little is known about the changes and mechanisms of autophagy in podocyte dysfunction under diabetic condition. In this study, we investigated the role of autophagy in podocyte biology and its involvement in the pathogenesis of diabetic nephropathy. Podocytes had a high basal level of autophagy. And basal autophagy inhibition either by 3-methyladenenine (3-MA) or by Beclin-1 siRNA was detrimental to its architectural structure. However, under diabetic condition in vivo and under high glucose conditions in vitro, high basal level of autophagy in podocytes became defective and defective autophagy facilitated the podocyte injury. Since the dynamics of endoplasmic reticulum(ER) seemed to play a vital role in regulating the autophagic flux, the results that Salubrinal/Tauroursodeoxycholic acid (TUDCA) could restore defective autophagy further indicated that the evolution of autophagy may be mediated by the changes of cytoprotective output in the ER stress. Finally, we demonstrated in vivo that the autophagy of podocyte was inhibited under diabetic status and TUDCA could improve defective autophagy. Taken together, these data suggested that autophagy might be interrupted due to the failure of ER cytoprotective capacity upon high glucose induced unmitigated stress, and the defective autophagy might accelerate the irreparable progression of diabetic nephropathy.

## Introduction

Diabetic nephropathy (DN) has become the most common cause of chronic kidney diseases (CKD) that ultimately progress to end-stage renal disease (ESRD) in many industrialized countries. And the most common clinical feature of diabetic nephropathy is progressive proteinuria due to compromised glomerular filtration barrier. The morphologically intricate podocyte and its slit diaphragm structure are of primary importance to the integrity of glomerular filtration barrier [1]. Over the past decades, numerous studies have pointed towards podocytopathy as the focal point of research in deciphering cellular and molecular mechanisms of diabetic nephropathy [2].

Podocytes, derived from embryonic precursor mesenchymal cells, are considered terminally differentiated cells in the mature kidney [3]. As highly differentiated neuron-like epithelial cells [4], podocytes have a very limited capacity for cell division and replenishment. Therefore, the ability to maintain homeostasis under certain pathophysiological stress seems to be very important in determining the fate of podocytes. Thus, finding better therapeutic targets to prevent podocyte injury in diabetic nephropathy has been a great challenge in understanding the modulating mechanisms of podocyte homeostasis.

Cellular homeostasis involves a balanced process of degradation and renewal of cytoplasmic components, including cellular organelles and proteins. Autophagy, a highly regulated lysosomal pathway involved in the recycling of cytosol and the removal of superfluous or damaged organelles, is essential for the survival, differentiation, development and homeostasis of cells [5]. Dysregulated autophagy has been suggested to play pathogenic roles in a variety of disease processes including cancer, neurodegeneration, diabetes, aging and heart disease [6]. Since autophagy is the only mechanism to degrade large structures, it plays an important role in the cellular refreshing which is particularly important in quiescent and terminally differentiated cells [7]. Therefore, considering its role in maintaining homeostasis [8], recently, the effect of autophagy in podocyte has garnered substantial attention. Hartleben et. al. have reported autophagy influences glomerular disease susceptibility and maintains podocyte homeostasis in aging mice [9]. However, as it stands, the changes and mechanisms of autophagy in podocyte dysfunction under diabetic condition and other diseases remain largely unknown. So the objective of this study is to investigate the significance of autophagy in podocyte injury and its involvement in the pathogenesis of diabetic nephropathy.

## Results

### Basal autophagy plays an essential role in maintaining podocyte biology and function

**Figure 1:**
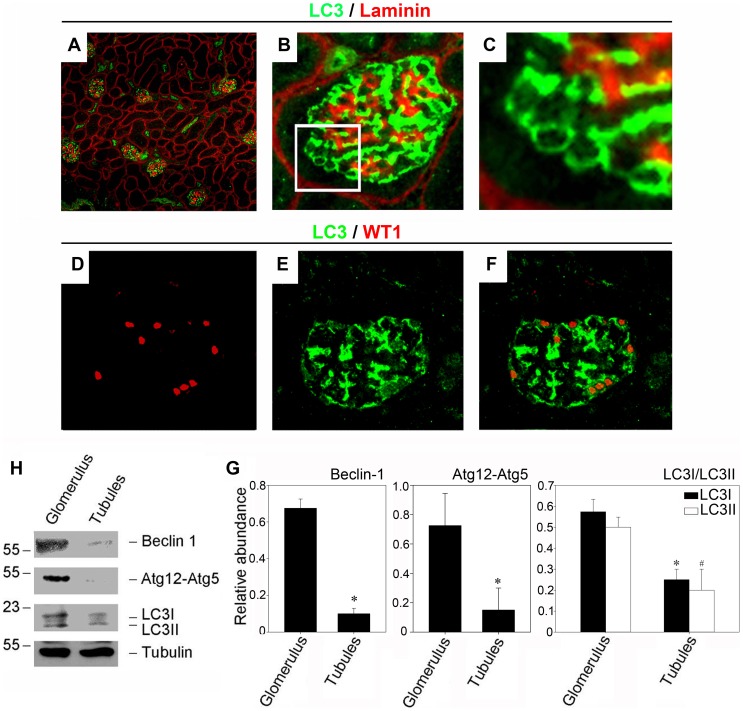
CD-1 mice exhibit high basal autophagy flux in glomerular podocytes. (A–C): Kidney section were immunostained with anti-LC3 antibody (green) to identify autophagosomes, followed by staining with anti-Laminin antibody (red) to sever as a marker for the basement membrane. (A): Low power (×100) field micrograph showing the localization of autophagosomes in the kidney. (B and C): High power (×400) field micrograph further demonstrated the autophagosomes (stained with the anti-LC3 antibody, green) were localized mainly on the epithelial side of the glomerular basement membrane (stained with the anti-Laminin antibody, red) where the podocytes reside. (D–F): Immunofluorescence staining demonstrates the occurrence of autophagy in podocytes, as illustrated by colocalization of WT-1 staining to recognize podocytes (D) and LC3 staining to identify autophagosomes (E). Merging the LC3 and WT-1 staining images is presented in F. (H and G): Western blot analysis shows a comparable protein abundance of autophagy related proteins such as Beclin-1, Atg12-5, and LC3 in the glomeruli lysates and the tubule lysates. The lysates were immunoblotted with Ab's against Beclin-1, Atg12-5, LC3 and α-tubulin, respectively. The relative abundances are presented in B after normalization with α-tubulin. * *P*<0.05 (n = 3).

To explore the significance of autophagy in the kidney diseases, we first investigated the distribution of autophagosomes in normal renal tissue by staining with anti-LC3 antibody. As shown in Figure 1, A through C, the distribution the autophagosomes (stained with the anti-LC3 antibody, green) were localized mainly on the epithelial side of the glomerular basement membrane (stained with the anti-Laminin antibody, red) where the podocytes reside. And the double immunofluorescence staining with WT-1 and LC3 further confirmed the occurrence of autophagy in podocytes (Figure 1 D through F). Western blot analysis also showed that the expression of autophagy related proteins such as Beclin-1, Atg12-Atg5 was evident in the glomeruli in Figure 1 (H and G).

**Figure 2:**
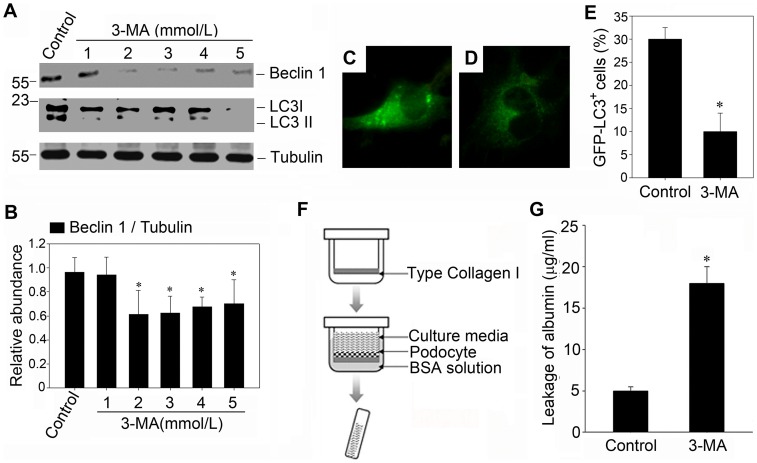
Inhibition of basal autophagy impairs the filtration barrier function of podocyte monolayer. (A): Western blot analysis confirms the inhibitory effect of 3-MA on autophagy in culture podocytes. Mouse podocytes were incubated with increasing amounts of 3-MA for 24 hours. (B): Graphical presentation shows the relative abundances of Beclin-1 after normalization with α-tubulin. Data are presented as mean ± SEM of three independent experiments. *P<0.05 vs. normal control; (C and D): Fluorescence staining of GFP-LC3 (400× magnification) in response to 3-MA treatment. Following transfection with GFP-LC3 plasmid as described in Materials and Methods, cells were treated without or with 3-MA (2 mmol/L) for 24 hours. (C): control group; (D): podocytes incubated with 3-MA for 24 hours. (E): Quantification of GFP-LC3 dotted cells after 3-MA treatment. A minimum of 100 GFP-LC3–transfected cells were counted. * *P*<0.05 vs. control; (F) Schematic depiction of the paracellular permeability influx assay. Podocyte monolayer on collagen-coated transwell filters was incubated without or with 3-MA (2 mmol/L) for 24 h, and albumin permeability across podocyte monolayer was then determined. (G): Graphic presentation of the albumin influx across the podocyte monolayer. Data are presented as mean ± SEM of three independent experiments. * *P*<0.05 vs. control.

**Figure 3:**
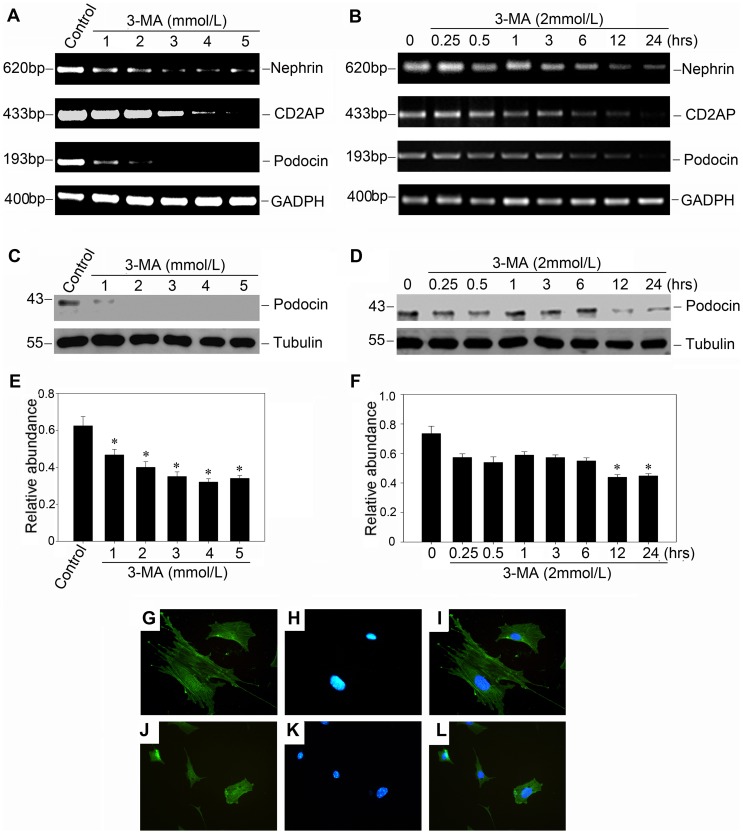
Autophagy inhibition by 3-MA suppresses the protein and mRNA expression of podocyte slit diaphragm proteins in a dose-dependent and time-dependent manner. (A) and (B): RT-PCR demonstrates that 3-MA (2 mmol/L) inhibited the mRNA expression of podocyte slit diaphragm proteins such as nephrin, CD2AP and podocin in a dose-dependent and time-dependent manner. Podocytes were incubated with either increasing amounts of 3-MA for 24 hours (A), or the same concentration of 3-MA (2 mmol/L) for various periods of time as indicated (B). (C) and (D): Western blot analysis shows that 3-MA (2 mmol/L) inhibited podocin protein expression in a dose- and time-dependent manner. Cell lysates were immunoblotted with Ab's against podocin and α-tubulin, respectively. (E) and (F): Quantitative determination of podocin protein abundance after normalization with α-tubulin. Data are presented as mean ± SEM of three independent experiments. *P<0.05 vs. normal control; (G–L): Immunofluorescence staining shows the localization of podocin in the control and 3-MA treated podocytes. (G–I): control group; (J–L): podocytes incubated with 3-MA (2 mmol/L) for 24 hours.

To investigate the role of basal autophagy in podocyte biology, we inhibited autophagy with 3-MA (Figure 2, A through E) and examined the filtration barrier function of podocyte by using a paracellular permeability influx. We found that 3-MA treatment could result in a greatly increased amount of albumin influx across the podocyte monolayer compared with the controls (Figure 2, F and G). Consistent with the result of paracellular permeability influx assay, RT-PCR analysis revealed that 3-MA could repress the mRNA expression of nephrin, CD2AP and podocin in both dose-dependent and time-dependent manner (Figure 3, A and B). Besides, western blot analysis also showed that the protein expression of podocin was repressed. Concomitant with the western blot alterations, as shown by immunofluorescence staining in Figure 3 (G through L), podocin protein was apparently crimpled around nucleolus after 3-MA treatment for 24 hours.

**Figure 4:**
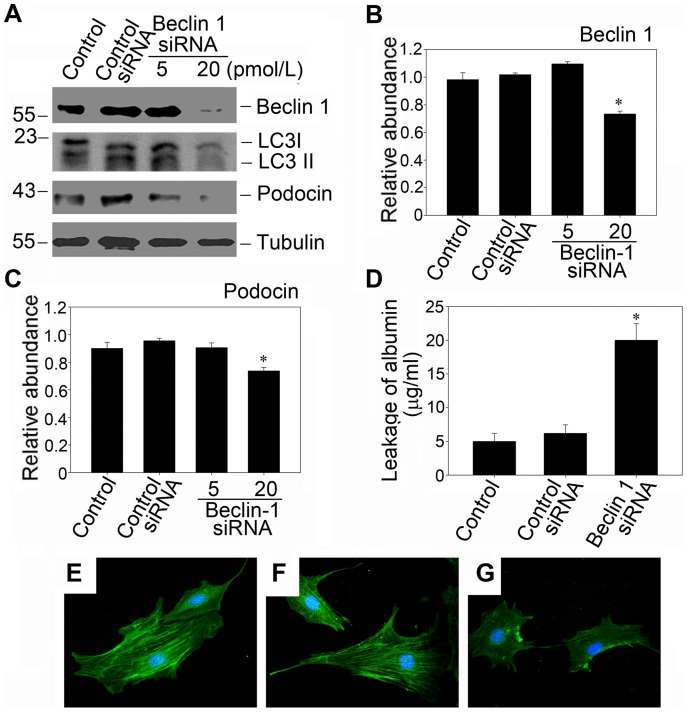
Autophagy inhibition by Beclin1 siRNA also decreases the expression of podocin and impairs the filtration barrier of podocyte monolayer. (A): Western blot analysis shows that beclin-1 silencing which was achieved by using 20 pmol/L siRNA significantly inhibited podocin protein expression. Mouse podocytes were transfected with beclin-1 siRNA and then incubated for 24 hours. (B and C): Quantitative determination of beclin-1 protein (B) and podocin protein (C) abundance after normalization with α-tubulin. Data are presented as mean ± SEM of three independent experiments. *P<0.05 vs. normal control; (D) Graphic presentation of the albumin influx across podocyte monolayer. Podocyts monolayer on collagen-coated Transwell filters was transfected with control siRNA or beclin-1 siRNA and then incubated for 24 hours, and albumin permeability across podocyte monolayer was determined. Values are means ± SEM; n = 3. *P<0.05 vs. control; (E–G): Representative photographs of podocin visualized by indirect immunofluorescence staining in the control, the control siRNA transfected cells and the beclin-1 siRNA transfected cells. (E): control; (F): podocytes transfected with control siRNA for 24 hours; (G): podocytes transfected with beclin-1 siRNA (20 pmol/L) for 24 hours.

To further confirm the association between autophagy inhibition and podocyte biology, we verified the inhibition of basal autophagy by silencing Beclin-1 gene, and the similar results were obtained (Figure 4). Inhibition of autophagy by Beclin-1 siRNA also repressed podocin expression (Figure 4, A and C) and resulted in a greatly increased amount of albumin influx (Figure 4D). These observations suggest that basal autophagy is essential in maintaining the architectural integrity of podocytes and the inhibition of basal autophagy may have severe negative impact on the filtration function of podocytes.

### Defective autophagy in podocytes from diabetic mice

**Figure 5:**
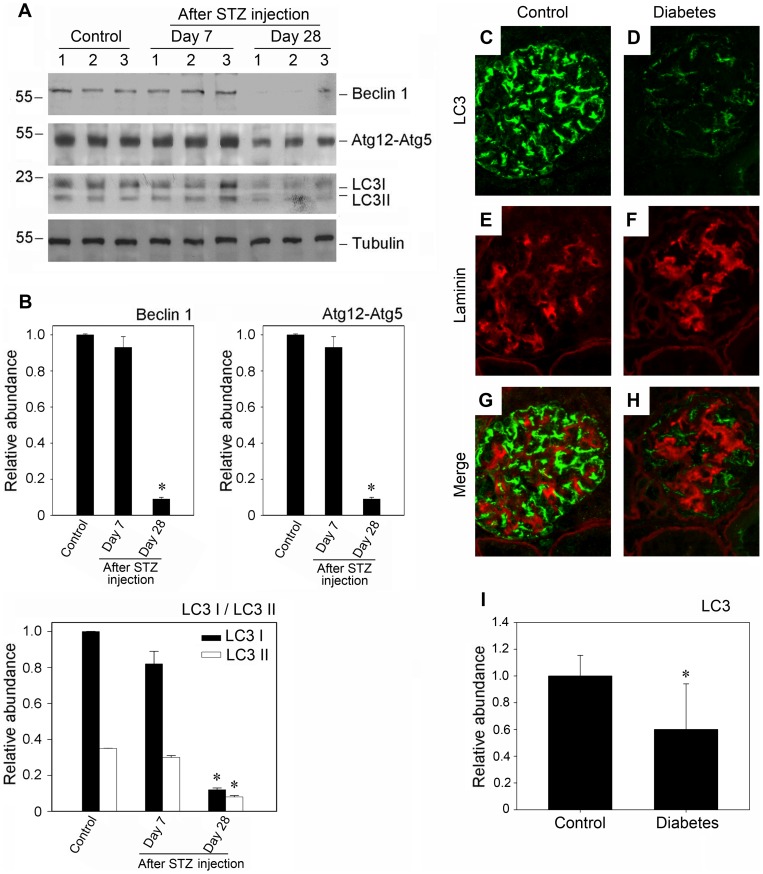
Suppression of autophagy in diabetic glomerular podocytes. (A) Western blot analysis shows the level of autophagy related proteins in diabetic glomerular. The glomerular lysates were blotted with specific antibodies against Beclin-1, Atg12-5 and LC3, respectively. Samples from two individual animals were used at each timepoint. (B) Quantitative determination of Beclin-1, Atg12-5 and LC3 protein abundance after normalization with α-tubulin. *P<0.05 (n = 3). (C–D) Immunofluorescence staining shows the changes of autophagosomes in the diabetic glomerular (400× magnification). Kidney section were immunostained with anti-LC3 antibody (green) to identify autophagosomes, followed by staining with anti-Laminin antibody (red) to sever as a marker for the basement membrane.(C, E and G): control group; (D, F and H): the group of 28 day diabetic mouse. (I): Quantification of LC3 immunofluorescence staining in nondiabetic glomeruli and diabetic glomeruli. 30 glomeruli were evaluated for each experimental animal (n = 5) through the middle part of the kidney. Glomeruli that did not have a glomerular tuft or that were sectioned close to the edge were disregarded. Data are presented as mean ± SEM. *P<0.05 vs. normal control.

To demonstrate the significance of autophagy in vivo under pathological conditions, we examined the changes of autophagy in diabetic nephropathy in mice. Figure 5A showed the change of autophagy in the progression of diabetic nephropathy as determined by Western blotting. As demonstrated with western blot analysis of glomerular lysates, the expression of autophagy related proteins such as Beclin-1, Atg12-Atg5 and LC3-II was markedly decreased in the course of diabetic nephropathy. As shown in Figure 5 (C through H), immunofluorescence staining also illustrated the diminished staining of LC3, coupled with the thickened staining of laminin in diabetic glomeruli. And similar salutary results were also obtained in the renal biopsies samples of diabetic patients (Figure S1). Hence, it seemed clear that the autophagy in podocytes became defective in the progression of diabetic nephropathy.

### High glucose inhibited high basal autophagy in podocytes

**Figure 6:**
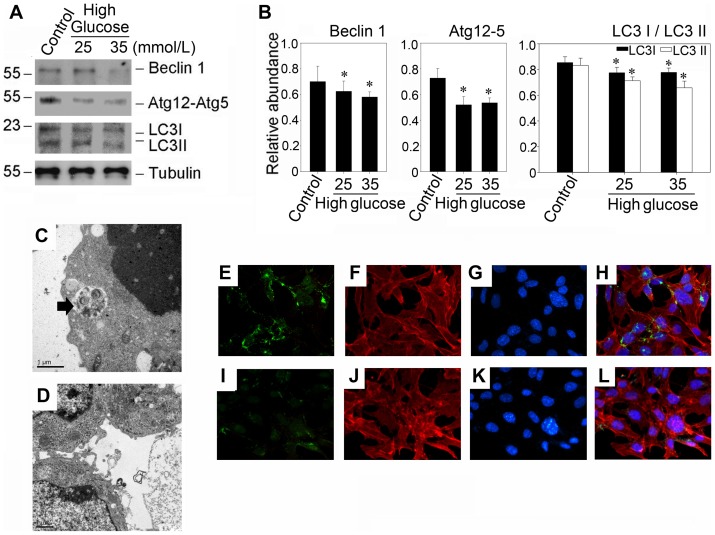
High glucose suppresses the expression of Beclin-1, Atg12-5 and LC3 in murine podocytes. (A). Western blot results demonstrated that high glucose (25 mmol/L and 35 mmol/L) suppresses the expression of Beclin-1, Atg12-5 and LC3. Murine podocytes were treated with high glucose (25 mmol/L and 35 mmol/L) for 48 hours. (B): Quantitative determination of the relative abundance of Beclin-1, Atg12-5 and LC3. Data are presented as mean ± SEM of three independent experiments. *P<0.05 vs. normal control; (C and D): Representative electronic micrographs shows autophagosomal structures in podocytes treated without or with high glucose (25 mM) for 48 hours. The arrows indicate autophagosomes. Bar 1 μm. (C): control group; (D): high glucose group incubated in 25 mmol/L D-glucose for 48 hours; (E–L): Double imunofluorescence staining shows the localization of LC3 (green, first column); β-actin (red, second column), and cell nucleus (blue, third column) in podocytes treated without or with high glucose (25 mM) for 48 hours. Merging of β-actin, LC3 and cell nucleus staining is presented in fourth column (H and L). (E, F, G and H): control group; (I, J, K and L): high glucose group incubated in 25 mmol/L D-glucose for 48 hours.

To investigate the change of autophagy under diabetic pathological stress, we examined the changes of autophagy related protein abundances after the treatment of high glucose which was considered as an initial trigger of diabetes. We found that podocytes expressed several autophagy related molecules, including Beclin-1, Atg5-Atg12 and LC3, indicating that basal autophagy existed under basal cultural condition. In light of the chronic condition of hyperglycemia under diabetic states, we assessed the expression of Beclin1, Atg5-12 and LC3 under sustained high glucose conditions. As illustrated in Figure 6 (B and C), western blot analysis revealed that the depressed expressions of autophagy related proteins had already been found in the presence of 25 mM and 35 mM high glucose at 48 hours, This result, together with the electron microscopic examination (Figure 6, D and E) and the immunofluorescence staining (Figure 6, F though M), indicated that sustained high glucose defected autophagy.

### Sustained high glucose induced autophagy deficiency facilitates the podocyte injury

**Figure 7:**
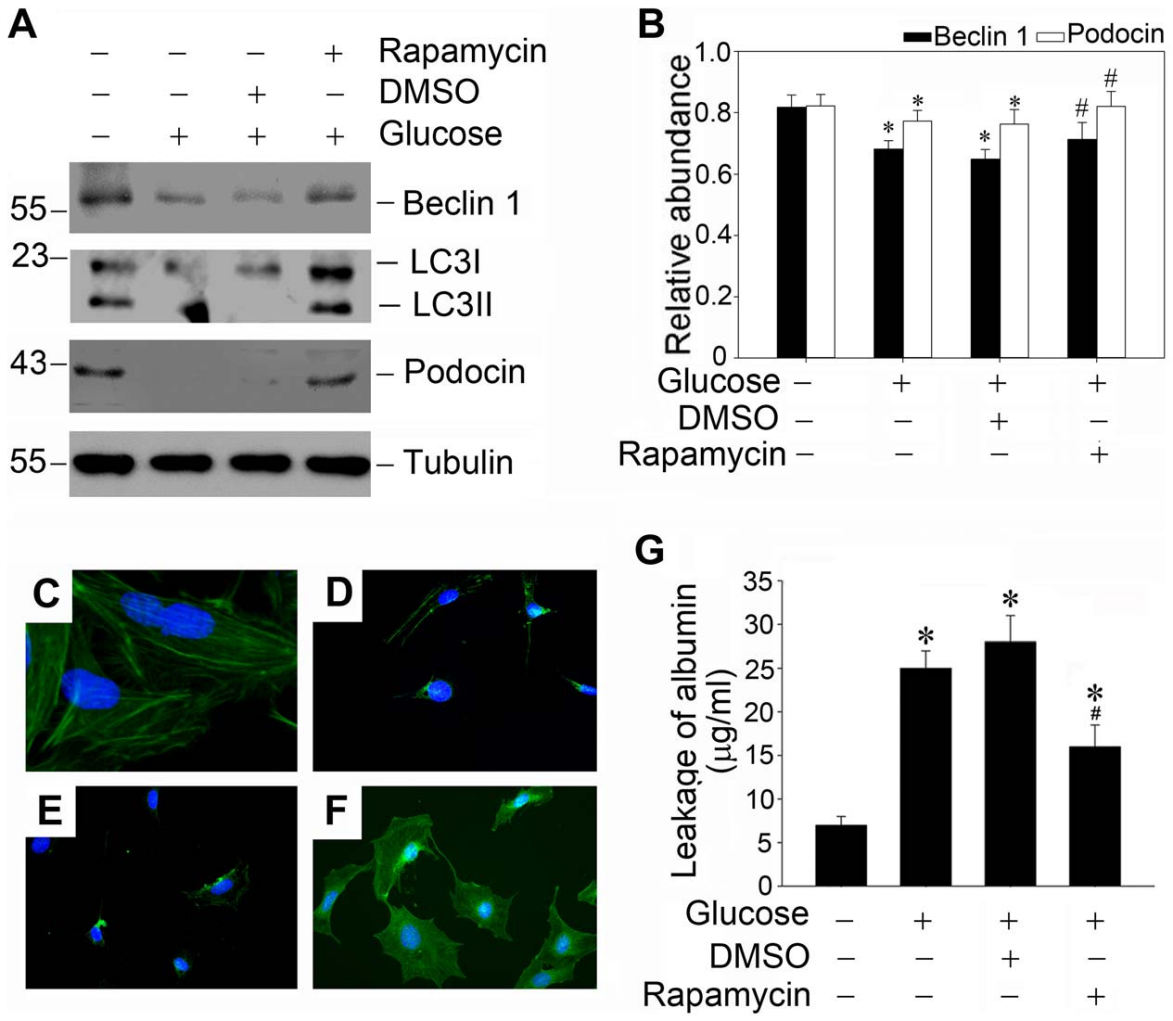
Rapamycin restores defective autophagy induced by high glucose and protects against high glucose induced podocyte injury. (A) Western blot analysis shows that low concentration of rapamycin (1 ng/ml) could restore defective autophagy induced by high glucose and protect against podocyte injury. Podocytes were pretreated with DMSO or 1 ng/ml rapamycin for 0.5 h, and followed by incubation with high glucose (25 mmol/L) for 48 hours. Whole-cell lysates were immunoblotted with specific antibodies against Beclin-1, LC3, podocin or α-tubulin, respectively. (B): Quantitative determination of Beclin-1 protein and podocin protein abundance after normalization with α-tubulin. Data are presented as mean ± SEM of three independent experiments. *P<0.05 vs. normal control; # *P*<0.05 vs. high-glucose-treated group. (C–F): Representative photographs of podocin visualized by indirect immunofluorescence staining in the control and treated podocytes as indicated. (C): control group; (D): high-glucose-treated group; (E): DMSO pretreated + high glucose treated group; (F): rapamycin pretreated + high glucose treated group; (G): Graphic presentation of the albumin influx across podocyte monolayer. Podocyte monolayer on collagen-coated transwell filters was incubated with various treatments as indicated for 48 hours. Data are presented as mean ± SEM of three independent experiments. * *P*<0.05 vs. normal control; # *P*<0.05 vs. high-glucose-treated group.

Since autophagy is essential in maintaining the architectural integrity of podocytes, autophagy deficiency at the later time points of sustained high glucose treatment seems to be involved in the high glucose induced podocyte injury. To investigate the role of autophagy deficiency, we used the autophagy enhancer rapamycin to restore autophagy. As shown in Figure 7 (A, B and G), the diminution of podocin expression and the impaired filtration barrier function were alleviated by restoring defective autophagy with low dose of rapamycin (1 ng/ml). And the filamentous pattern of podocin distribution was also improved by the autophagy enhancer rapamycin as shown by double immunofluorescence staining (Figure 7, C through F). These data insinuated that defective autophagy may be pivotal in the irreparable progression of podocyte injury and restoring autophagy could be a new target for remission of podocyte injury.

### The endoplasmic reticulum stress mediates the high glucose induced defective autophagy

**Figure 8:**
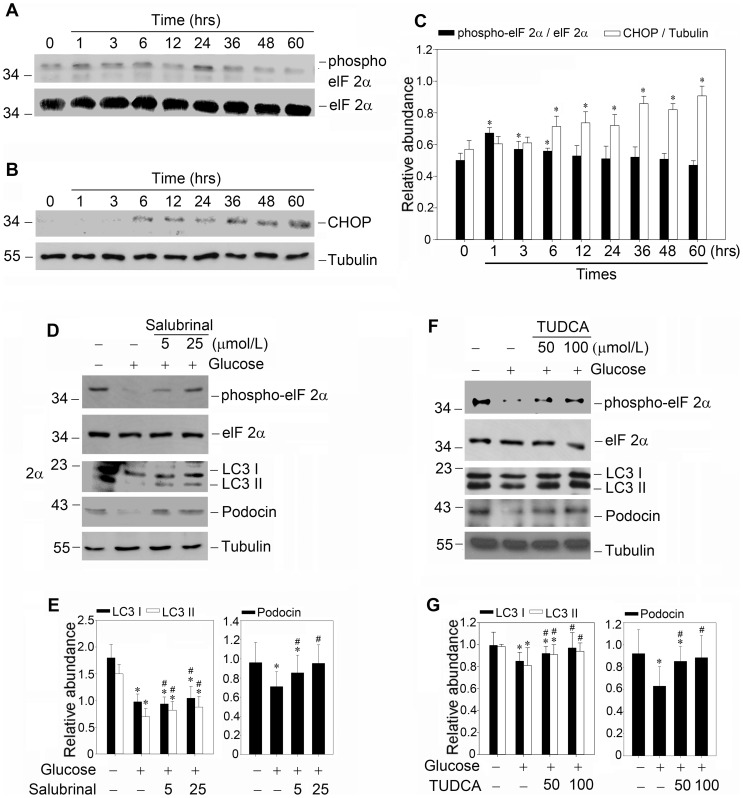
Enhancement of cytoprotective output of high glucose induced endoplasmic reticulum stress could restore defective autophagy. (A and B): Kinetics of elF2α and CHOP in high glucose induced endoplasmic reticulum stress. Murine podocytes were treated with high glucose (25 mmol/L) for the indicated times, and elF2α phosphorylation and CHOP expression were detected by immunoblotting; (C): Quantitative determination of the phosphorylation of elF2α and CHOP protein abundance after normalization with α-tubulin. Data are presented as mean ± SEM of three independent experiments. *P<0.05 vs. control; (D): Salubrinal could restore high glucose suppressed autophagy and podocin expression. Podocytes were pretreated with salubrinal for 0.5 h, followed by incubation with high glucose (25 mmol/L) for 48 hours. Whole-cell lysates were immunoblotted with specific antibodies against phosphorylated elF2α, elF2α, LC3, podocin or α-tubulin, respectively. (F): Taurine-conjugated ursodeoxycholic acid (TUDCA) also restores high glucose suppressed autophagy and podocin expression. Podocytes were pretreated with TUDCA for 0.5 h, followed by incubation with high glucose (25 mmol/L) for 48 hours. (E and G): Quantitative determination of the relative abundance of LC3 and podocin among different groups. Data are presented as mean ± SEM of three independent experiments. *P<0.05 vs. normal control; # *P*<0.05 vs. high-glucose-treated group.

Since growing evidence has indicated that ER is the primary source supplying membranes for autophagosome biogenesis, we reasoned that the dynamics of ER may play a vital role in regulating the autophagic flux. With respect to this hypothesis, we first examined the expression of ER stress proteins after high glucose treatment, such as the phosphorylation of eIF2α and the expression of CHOP. As shown in Figure 8A, high glucose caused eIF2α phosphorylation as early as 1 hour and the phosphorylation then gradually subsided from 36 to 60 hours. However, the expression of the proapoptotic transcription regulator CHOP still maintained at an elevated level at the same period of time as shown in Figure 8, B and C. As mentioned by Lin JH [10], this may be due to the time-related switches in the endpoint of the unfolded protein response (UPR) manifested by selective attenuation of cytoprotective unfolded protein response (UPR) output coupled with sustained CHOP production. Based on our observations above and the relationship between ER stress and autophagy, it is conceivable to speculate that autophagy may be the downstream of the cytoprotective UPR output. Since eIF2α has been proved to be the upstream factor of autophagy, we used salubrinal, an agent which acts as a selective inhibitor of eIF2α dephosphorylation [11] to restore eIF2α phosphorylation at the later time points of high glucose treatment. As shown in Figure 8D, we found that salubrinal treatment could restore defective autophagy and improve high glucose induced diminution of podocin expression. We also applied taurine-conjugated ursodeoxycholic acid (TUDCA) to enhance the cytoprotective capacity of ER and evaluated its effects. Intriguingly, similar salutary results were obtained (Figure 8, F and G). These observations suggested that the prolonged high glucose condition may exhaust the cytoprotective output of ER stress which might play an important role in mediating autophagy flux. Defective autophagy, which was known to decrease with age or other diseases, might be not just an innocent bystander, and it might amplify the proapoptotic output of ER stress and participated in the podocyte injury.

### Taurine-conjugated derivative (TUDCA) ameliorates diabetic nephropathy through restoring autophagy flux in mice

**Figure 9:**
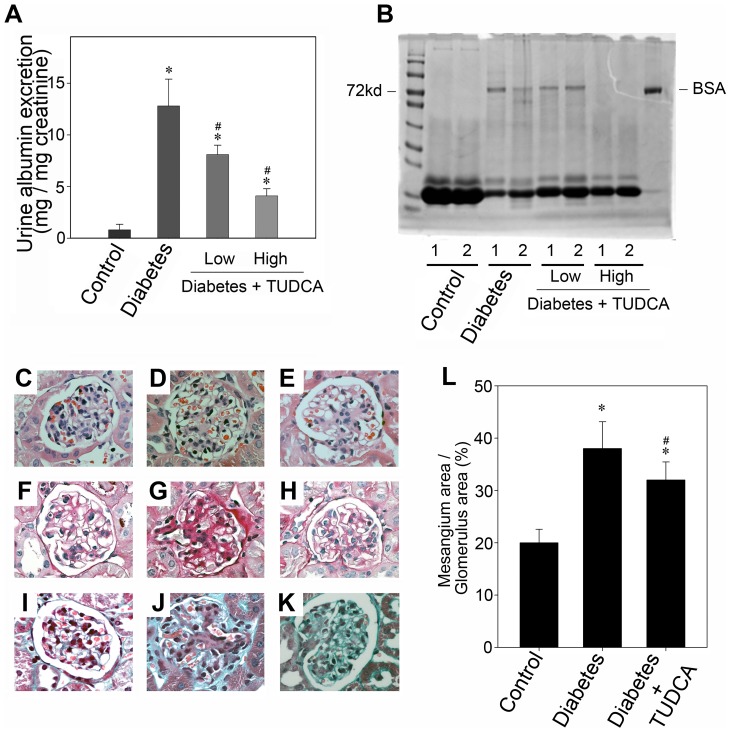
TUDCA attenuates albuminuria and improves histopathological lesion in diabetic mice. (A) TUDCA attenuates albuminuria in diabetic mice. Shown is graphic presentation of urinary albumin/creatinine ratio. Data are presented as means ± SEM of three experiments. n = 6; * P<0.05 vs. normal control. # *P*<0.05 vs. the group of 28 day diabetic mouse. (B) Representative SDS-PAGE shows the urine proteins in different groups of mice as indicated. Numbers (1 and 2) denote each individual animal in a given group. (C–K) The light microscopic appearance of representative glomeruli (400× magnification) is shown stained with H&E (C–E), PAS (F–H), and Masson's trichrome (I–K). The left column (C, F and I): control group; The second column (D, G and J): diabetic group; The third column (E, H and K): diabetic group treated with 500 mg/kg/day TUDCA. (L): Quantification of extracellular mesangial matrix area in relation to glomerular tuft area. Results are expressed as average percentage of glomerular area occupied by the mesangial matrix. 30 glomeruli were evaluated for each experimental animal (n = 6) through the middle part of the kidney. * P<0.05 vs. normal control. # *P*<0.05 vs. the group of 28 day diabetic mouse.

**Figure 10:**
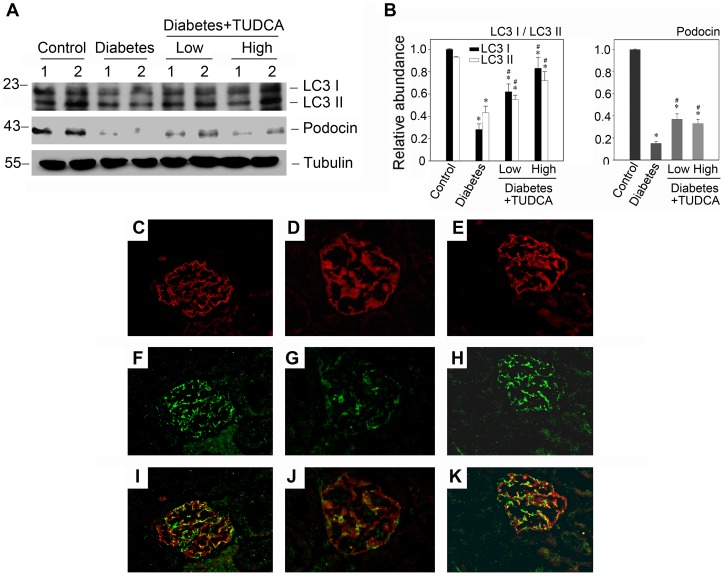
TUDCA restores the suppressed autophagy in diabetic mice and attenuates podocyte injury. (A) Western blot analysis demonstrates an improvement of autophagy level and podocin expression in the glomeruli isolated from mice as indicated. The glomerular lysates (made from the pool of kidneys from six animals/group) were separated on a SDS-polyacrylamide gel and immunoblotted with a specific monoclonal antibody against LC3, podocin and α-tubulin, respectively. Samples from two individual animals were used at each timepoint. (B) Quantitative determination of LC3 and podocin protein abundance after normalization with α-tubulin. Data are presented as means ± SEM of three experiments. n = 6, *P<0.05 vs. normal control. #*P*<0.05 vs. diabetic group. (C–K) Immunofluorescence staining shows the changes of autophagosomes in various groups (400× magnification). Kidney section were immunostained with anti-LC3 antibody (green) to identify autophagosomes, followed by staining with anti-podocin antibody (red) to sever as a marker for podocytes. The left column (C, F and I): control group; The second column (D, G and J): diabetic group; The third column (E, H and K): diabetic group treated with 500 mg/kg/day TUDCA.

As mentioned above, TUDCA is a chemical chaperone which can enhance the cytoprotective capacity of the ER [12]. And we also had proven it was able to restore defective autophagy and impede the progression of high glucose induced podocyte injury in vitro, so we next examined the effects of TUDCA in the progression of diabetic nephropathy in vivo. Although the blood glucose level of TUDCA treated group appeared to be lower than vehicle treated group, they were all higher than the diagnostic criteria of diabetes or random blood glucose level, and there were no statistically significant difference between both diabetic groups (Table S1). Morphology was evaluated by hematoxylin and eosin (H&E), periodic acid-Schiff (PAS), and Masson's Trichrome Staining. Along with the remission of endoplasmic reticulum stress (as shown in Figure S2), the mesangial matrix expansion in diabetic glomeruli, accompanied by basement membrane thickening, could be improved by TUDCA (Figure 9, C through K). And the urine albumin levels were also ameliorated by TUDCA dramatically (Figure 9, A and B). Besides, western blot analysis showed that the autophagy level could be restored with the treatment of TUDCA in vivo (Figure 10). Accompanying with the autophagy restoration, the podocin expression was improved (Figure 10, A and B). Concomitant with the western blot alterations, as shown in Figure 10, C through K, double immunofluorescence staining for podocin (red) and LC3 (green) also demonstrated that the diffuse distribution of podocin and the diminished LC3 staining could be partly restored by TUDCA. These observations implied that the ER cytoprotective enhancer TUDCA could restore the defective autophagy and podocyte injury in the progression of diabetic nephropathy.

## Discussion

Given the increasing prevalence of diabetes in both developed and developing countries, it is likely that its chronic microvascular complication diabetic nephropathy (DN) has become the single most important factor leading to end-stage renal disease (ESRD) in the world. Diabetic nephropathy is clinically characterized by the early increase in albuminuria and progressive proteinuria which not only predicts the outcomes of ESRD but also implies the damage of glomerular filtration barrier [13]. Podocytes, together with glomerular endothelial cells and glomerular basement membrane, form the glomerular filtration barrier. The diseases of podocytes are mainly presented with proteinuria [14]. Therefore, podocytopathies have become the focal point of research in deciphering molecular mechanisms of DN over the past decade [2]. Since podocytes are terminally differentiated cells similar to neurons and have a very limited capacity for division and replacement, it is conceivable to speculate that there are potential reparative mechanisms for podocytes to maintain homeostasis under different physiological and pathological stresses. A comprehensive understanding of the molecular mechanisms in modulating homeostasis could provide new therapeutic targets for prevention and remission of diabetic nephropathy.

Cellular homeostasis involves a constant balance between biosynthesis and degradative processes. In eukaryotic cells, there are two major protein degradation systems: one is the ubiquitin-proteasome system; the other is the lysosomal system [15]. Autophagy, which was first coined by Christian de Duve in 1963, is an evolutionarily conserved lysosomal pathway mostly implicated in long-lived proteins and damaged organelles [16]. When autophagy is induced, the isolation membrane called phagophore expands to form the autophagosome, a double-membraned vesicle that sequesters the cytoplasmic materials or damaged organelles. After the autophagosome-lysosome fusion, the sequestered components are degraded by lysosomal hydrolases [17]. In this way, autophagy may help the cell modify the protein components to respond to extrinsic stimuli and maintain homeostasis [18]. So far, one of the most inspiring discoveries in the research of autophagy is that the loss of autophagy in the central nervous system can result in neurodegeneration [19], [20]. Since the neurons and the podocytes have so many similarities both morphologically and biochemically [4], it is most likely that podocyte is also a preferential target for exploring autophagy abnormalities. In accordance with the previous study conducted by Asanuma [21] and Hartleben [22], our data also confirms that podocytes have a higher level of constitutive autophagy than other intrinsic renal cells, and inhibition of basal autophagy either by 3-methyladenenine (3-MA) or by Beclin-1 siRNA is detrimental to podocytès architectural structure and cell viability. Thus, autophagy seems to be a well-established contributor to podocyte homeostasis, and either enhanced [23] or dysregulated autophagy [22] might involve in podocyte injury. However, since most of the studies were conducted using transgenic or gene knock-out mice, little is known about the evolution of autophagy and the underlying mechanisms in the process of diabetic nephropathy and other kidney diseases. Here, we provide in vitro and in vivo evidence to support that sustained high glucose ultimately defects autophagy which participates in the relentless progression of podocyte injury. Since defective autophagy seems to be not just an innocent bystander, our findings are of clinical interests. The understanding of its underlying mechanisms may identify novel avenues for prevention and treatment diabetic nephropathy.

The endoplasmic reticulum (ER) is the most important organelle for protein synthesis, folding and maturation in the eukaryotic cell. Intracellular proteins transit through this compartment and only properly folded proteins are allowed to leave the ER while misfolded proteins are degraded [24], [25]. To maintain fidelity, the ER has a strict quality-control system for protein proof-reading and disposal (22). Under ER stress conditions, too many unfolded or misfolded proteins are accumulated in the ER lumen. Only if the adaptive capacity fails to reestablish the homeostasis, ER stress may result in the progression of diseases [26]. And it has been documented that ER stress involves in a variety of diseases such as diabetes, ischemia and neurodegenerative disorders. Recently, two groups have demonstrated a direct physical connection between the ER and newly formed phagophore [27], [28]. The ER was claimed to be the main contributor to the autophagosomes. So we believe that the dynamics of ER plays a vital role in regulating the autophagic flux. In addition, as we mentioned above, autophagy is a bulk degradation system that can degrade all forms of misfolded proteins whereas proteasomal degradation is limited to soluble ones. Growing evidence has indicated that autophagy plays an important role in the ER quality control system [29], although the ubiquitin proteasome system was originally thought to be endoplasmic reticulum associated degradation (ERAD) [30], [31]. However, the direct link between ER stress and autophagy was only reported about five year ago [32]. Studies on ER stress and autophagy markers in podocytes and podocyte diseases are limited, and many questions concerning the signaling pathways linking ER stress to autophagy remain largely unanswered [33]. There are three major arms of ER stress [34]: protein kinase RNA (PKR)-like ER kinase (PERK), inositol-requiring protein-1 (IRE1), and activating transcription factor-6 (ATF6) pathways, of which eIF2α has been accepted as the upstream factor of autophagy [35]. Our studies found that high glucose could induce eIF2α phosphorylation, however, at the later time points, prolonged treatment might cause eIF2α dephosphorylation while the proapoptotic CHOP expression remained elevated, indicating the time-related switches of the cytoprotective output and proapoptotic output in the endpoint of the unfolded protein response (UPR) [11]. Once eIF2α dephosphorylation was selectively inhibited by salubrinal, the high glucose induced defective autophagic flux and podocyte injury could be relieved considerably. These data suggested that high glucose induced defective autophagic flux might be a result of the exhaustion of the cytoprotective output and enhancing cytoprotective capacity by salubrinal might restore high glucose induced defective autophagy. To further confirm the role of autophagy in the cytoprotective output of ER stress, we assessed the effect of chemical chaperone taurine-conjugated ursodeoxycholic acid (TUDCA), which can enhance the cytoprotective capacity of the ER [36]. TUDCA can improve defective autophagy and podocyte injury not only in vitro but also in vivo. Since recent studies have suggested that the autophagy response might serve a cytoprotective function challenged with ER stress [37], these observations might offer a rationale that the change of autophagy may be mediated by the switch of cytoprotective output during ER stress [10]. Upon unmitigated ER stress, the process of autophagy is disturbed and defected as a result of the exhaustion of cytoprotective output. This in turn, because of the defective autophagy, the quality-control system fails to reestablish the ER homeostasis, and the high glucose induced ER stress may result in the progression of diabetic nephropathy.

In summary, our studies suggested that autophagy might play an essential role in maintaining the homeostasis of podocyte as an intrinsic endoplasmic reticulum-associated degradation (ERAD) system. However, upon unmitigated stress, autophagy could be interrupted due to the failure of ER cytoprotective capacity [10]. Defective autophagy may elicit overwhelming progression of podocyte injury and in turn leads to diabetic nephropathy. In view of the role of autophagy, it is reasonable to speculate that restoring ER cytoprotective capacity and defective autophagy may hold promise as novel avenues for preventing and halting the progression of diabetic nephropathy.

## Materials and Methods

### Ethics statement

All of the following details of the study were approval by institutional review board of Nanjing Medical University.

### Animal model

Male CD-1 mice weighed ∼18 to 22 grams were acquired from the Specific Pathogen-Free (SPF) Laboratory Animal Center of Nanjing Medical University. According to the guidelines of the Institutional Animal Care and Use Committee of the National Institutes of Health at Nanjing Medical University, animals were treated humanely and rendered diabetic by daily intraperitoneally injections of 50 mg/kg streptozotocin (STZ, Sigma, St. Louis, MO) for 5 days. For the sham-operated group, normal saline was administered. Tail blood glucose (TBG) levels were monitored consecutively and diabetic status was confirmed by the manifestation of weight loss, polyuria, and TBG level greater than 500 mg/dl. The diabetic mice were sacrificed at 1, 2 and 4- weeks after the treatment; serum and urine were collected and the kidneys were harvested. Glomeruli were isolated from one portion of the kidney by a graded sieving procedure as described previously [38], while the remaining kidney was prepared for histological studies.

To evaluate the effect of TUDCA therapy on the podocyte injury and the autophagic level changes in diabetic mice, the diabetic mice were randomly assigned into three groups: diabetic mice to be treated with normal saline, diabetic mice to be treated with 250 mg/kg/day tauroursodeoxycholic acid (TUDCA, Sigma, St. Louis, MO) and diabetic mice to be treated with 500 mg/kg/day TUDCA as previously described [12]. Mice were sacrificed at 4- weeks after the treatment; serum and urine were collected and the kidneys were harvested. Glomeruli were isolated from one portion of the kidney by a graded sieving procedure as described previously [38], while the remaining kidney was prepared for histological studies. The protocol was approved by the Committee on the Ethics of Animal Experiments of Nanjing Medical University (Permit Number: KY2012018). All efforts were made to minimize animal suffering and to reduce the number of animal used.

### Urinary Albumin and Creatinine Assay

Urinary albumin was measured by using a mouse Albumin ELISA Quantitation Kit according to the manufacturer's protocol (Bethyl Laboratories, Inc., Montgomery, TX). Urinary creatinine was determined by a routine procedure as described previously [39].

### Cell culture and treatment

The conditionally immortalized mouse podocyte cell line was kindly provided by Dr. Zhihong Liu (Research Institute of Nephrology, Nanjing General Hospital of Nanjing Military Command, Nanjing, Jiangsu Province, China) while their cell lines were provided by Dr Peter Mundel (Mount Sinai School of Medicine, New York, NY, USA) and described previously [40]. Cells were cultured at the permissive temperature (33°C) in RPMI-1640 medium supplemented with 10% fetal bovine serum (Invitrogen, Grand Island, NY) and recombinant interferon-γ (Invitrogen, Carlsbad, CA, USA). To induce differentiation, podocytes were grown under nonpermissive conditions at 37°C for 10–14 days in the absence of interferon-γ. After serum starvation for 16 h, cells exposed to the treatment for the indicated time periods.

### Western immunoblot analysis

Immortalized mouse podocytes were lysed with SDS sample buffer (62.5 mmol/L Tris·HCl, pH 6.8, 2% SDS, 10% glycerol, 50 mM DTT, and 0.1% bromophenol blue). And kidney tissues were homogenized by a polytron homogenizer (Brinkmann Instruments, Westbury, NY) in RIPA lysis buffer (1% NP-40, 0.1% SDS, 100 ug/ml PMSF, 0.5% sodium deoxycholate, 1 mmol/L sodium orthovanadate, 2 ug/ml aprotinin, 2 ug/ml antipain, and 2 ug/ml leupeptin in PBS) on ice. The supernatants were collected after centrifugation at 16,000 *g* at 4°C for 30 min. Protein concentration was determined using a BCA protein assay kit (Sigma, USA), and whole tissue lysates were mixed with an equal amount of 2×SDS loading buffer (125 mmol/L Tris-HCl, 4% SDS, 20% glycerol, 100 mmol/L DTT, and 0.2% bromophenol blue). Samples were heated at 100°C for 5∼10 min before loading and were separated on precast 10 or 12% SDS polyacrylamide gels (Bio-Rad, Hercules, CA). Detection of protein expression by Western blotting was carried out according to the established protocols. The primary antibodies used were as follows: anti-podocin (P0372, Sigma-Aldrich), Anti-α-tubulin (T6074, Sigma-Aldrich), anti-Beclin-1 (sc-48381, Santa Cruz Biochemical), anti-Atg12 (sc-68884, Santa Cruz Biochemical), anti-LC3 (no.2775S, Cell Signaling), anti-CHOP (sc-7351, Santa Cruz Biochemical), anti-phospho-eIF2 alpha (sc-101670, Santa Cruz Biochemical) and anti-total eIF2 alpha (sc-133132, Santa Cruz Biochemical). Quantification was performed by measurement of the intensity of the signals with the aid of the National Institutes of Health Image software package.

### RT-PCR analysis

Total RNA was prepared using a TRIzol RNA isolation system according to the instructions specified by the manufacturer (Invitrogen, Grand Island, NY)). The first strand of cDNA was synthesized using 2 ug of RNA in 20 ul of reaction buffer using Moloney leukemia virus-RT (Promega, Madison, WI) and random primers at 42°C for 30 min. PCR was performed using a standard PCR kit on 1 ul aliquots of cDNA and HotStarTaq polymerase (Promega) with specific primer pairs. The sequences of primer pairs were as follows: Nephrin (forward) 5′-CCC AAC ACT GGA AGA GGT GT-3′ and (reverse) 5′-CTG GTC GTA GAT TCC CCT TG-3′; CD2AP (forward) 5′-CGA GTT GGG GAA ATC ATC AG-3′ and (reverse) 5′-TGA GGT AGG GCC AGT CAA AG-3′;Podocin (forward) 5′-AGA CTC TCC CAT GTT ATA GG-3′ and (reverse) 5′-TAT AGT GAT TCT CCC TCA GAT-3′; GADPH (forward) 5′-CCA TGT TCG TCA TGG GTG TGA ACC A-3″and (reverse) 5′-GCC AGT AGA GGC AGG GAT GAT GTT C-3′. The PCR products were size fractionated on a 1.0% agarose gel and detected by NA-green (D0133, Beyotime) staining.

### GFP-LC3 transfection

Podocytes were plated at a density of 2×10^5^ on glass cover slips in six-well plates and cultured up to 70% confluence. Transient transfections with GFP-LC3 plasmid (kindly provided by Dr. Wenxing Ding, Department of Pharmacology, Toxicology & Therapeutics, University of Kansas Medical Center) were carried out by using Lipofectamine 2000 (Invitrogen, Carlsbad, CA) as per manufacturer's recommendation. Microphotographs of GFP-LC3 fluorescence were obtained with a fluorescence microscope. The detection of punctated staining of GFP-LC3 from the diffuse staining indicated the formation of autophagosomes.

### Immunofluorescence staining

Indirect immunofluorescence staining was performed according to an established procedure. Briefly, cells cultured on coverslips were washed twice with cold PBS and fixed with cold methanol/acetone (1∶1) for 10 min at −20°C. Following three extensive washings with PBS, the cells were treated with 0.1% Triton X-100 for 5 min and blocked 2% normal donkey serum in PBS buffer for 40 min at room temperature and then incubated with the specific primary antibodies described above, followed by staining with FITC or TRITC-conjugated secondary antibody. Cells were stained with 4′,6- diamidino-2-phenylindole HCl to visualize the nuclei. Slides were viewed with a Nikon Eclipse 80i Epi-fluorescence microscope equipped with a digital camera (DS-Ri1, Nikon). In each experimental setting, immunofluorescence images were captured with identical light exposure times.

### Albumin Influx Assay

A simple albumin influx assay was used to evaluate the filtration barrier function of podocyte monolayer as described previously [41]. Briefly, podocytes (5×10^3^) were seeded onto the collagen-coated transwell filters (3 μm pore; Corning, New York, NY) in the top chamber and cultured under differentiating conditions. After 10 days, podocytes were serum-starved overnight and treated as indicated for various periods of time. Cells were washed twice with PBS supplemented with 1 mmol/L MgCl_2_ and 1 mmol/L CaCl_2_ to preserve the cadherin-based junctions. The top chamber was then refilled with 0.15 ml of RPMI 1640 and the bottom chamber with 1 ml of RPMI 1640 supplemented with 40 mg/ml of bovine serum albumin and incubated at 37°C. A small aliquot of media from the top chamber was collected 6 hours later and the albumin concentration was determined using a bicinchoninic acid protein assay kit (Sigma).

### Electron Microscopy

Cells were harvested gently using Trypsin EDTA, washed with PBS, fixed in 2% glutaraldehyde with 0.2% tannin in PBS, postfixed in osmium tetroxide, and embedded in Epon. Sections were cut at 80 nm with an ultramicrotome (Ultracut E, Leica). The specimens were examined with a Tecnai G2 Spirit BioTWIN electron microscope and photographed with an AMT 2k CCD camera at the Department of Pathology of Nanjing Jinling Hospital.

### Statistical analysis

Western blotting, RT-PCR, and immunofluorescence staining were all repeated at least three times independently. For the histologic analysis and immunostaining, quantification was performed by using Image-Pro Plus 6.0 software. For Western blot analysis, quantitation was performed by scanning and analyzing the intensity of the hybridization signals using NIH Imagine software. Statistical analysis was performed using Sigma Stat software (Jandel Scientific Software, San Rafael, CA). Comparisons between groups were made using one-way ANOVA, followed by Student's *t-*test. *P*<0.05 was considered significant.

## Supporting Information

Figure S1 The autophagy marker LC3 was downregulated in diabetic glomerular podocytes in the renal biopsies. (A–F) Representative micrographs by immunofluorescence staining of LC3 (green) and Laminin (red) in nondiabetic patients and diabetic patients, demonstrating the suppression of autophagy in diabetic renal biopsies. G: The relative abundance of the semiquantitative histomorphometric analysis of the immunostaining of LC3. The patients (n = 3) with mild mesangium proliferative glomerulonephritis whose proteinuria were about 0.32±0.174 g/24 h were choosen as a control group; The diabetic patients (n = 3) whose proteinuria were about 5.21±1.863 g/24 h were choosen as a diabetes group. For each renal biopsy specimen, 10 glomeruli were evaluated by using Image-Pro Plus 6.0 software. Data are presented as mean ± SEM. *P<0.05 vs. control.
(TIF)

Figure S2 TUDCA attenuates ER stress in diabetic glomerular podocytes. (A–C) Representative micrographs by immunohistochemical staining of CHOP. A: control group; B: diabetic group; C: diabetic group treated with 500 mg/kg/day TUDCA. (D–F) Representative micrographs by immunohistochemical staining of GRP78. D: control group; E: diabetic group; F: diabetic group treated with 500 mg/kg/day TUDCA. G: The relative abundance of the semiquantitative histomorphometric analysis of the immunostaining of CHOP and GRP78. 30 glomeruli were evaluated for each experimental animal (n = 6). Data are presented as mean ± SEM. * P<0.05 vs. normal control. # *P*<0.05 vs. the group of 28 day diabetic mouse.
(TIF)

Table S1 Characteristics of the diabetic mice treated without or with TUDCA.
(DOC)
