# Supplementary figures and images for: Autophagy Attenuates Diabetic Glomerular Damage through Protection of Hyperglycemia-Induced Podocyte Injury
Source: PLoS One. 2013 Apr 11;8(4):e60546. doi: 10.1371/journal.pone.0060546 (PMC3623813; doi:10.1371/journal.pone.0060546)

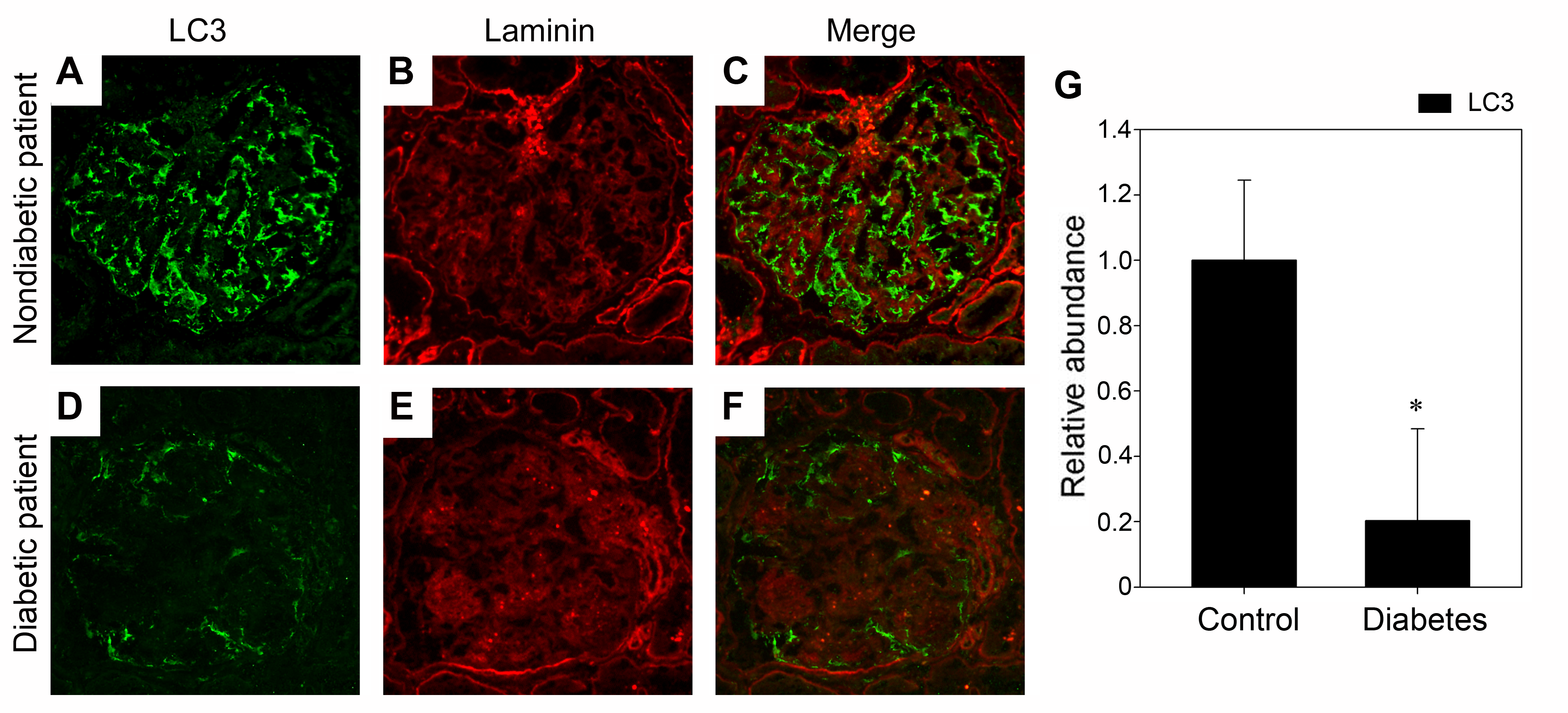

Supplement: Figure S1 — The autophagy marker LC3 was downregulated in diabetic glomerular podocytes in the renal biopsies. (A–F) Representative micrographs by immunofluorescence staining of LC3 (green) and Laminin (red) in nondiabetic patients and diabetic patients, demonstrating the suppression of autophagy in diabetic renal biopsies. G: The relative abundance of the semiquantitative histomorphometric analysis of the immunostaining of LC3. The patients (n = 3) with mild mesangium proliferative glomerulonephritis whose proteinuria were about 0.32±0.174 g/24 h were choosen as a control group; The diabetic patients (n = 3) whose proteinuria were about 5.21±1.863 g/24 h were choosen as a diabetes group. For each renal biopsy specimen, 10 glomeruli were evaluated by using Image-Pro Plus 6.0 software. Data are presented as mean ± SEM. *P<0.05 vs. control. (TIF) [file pone.0060546.s001.tif]

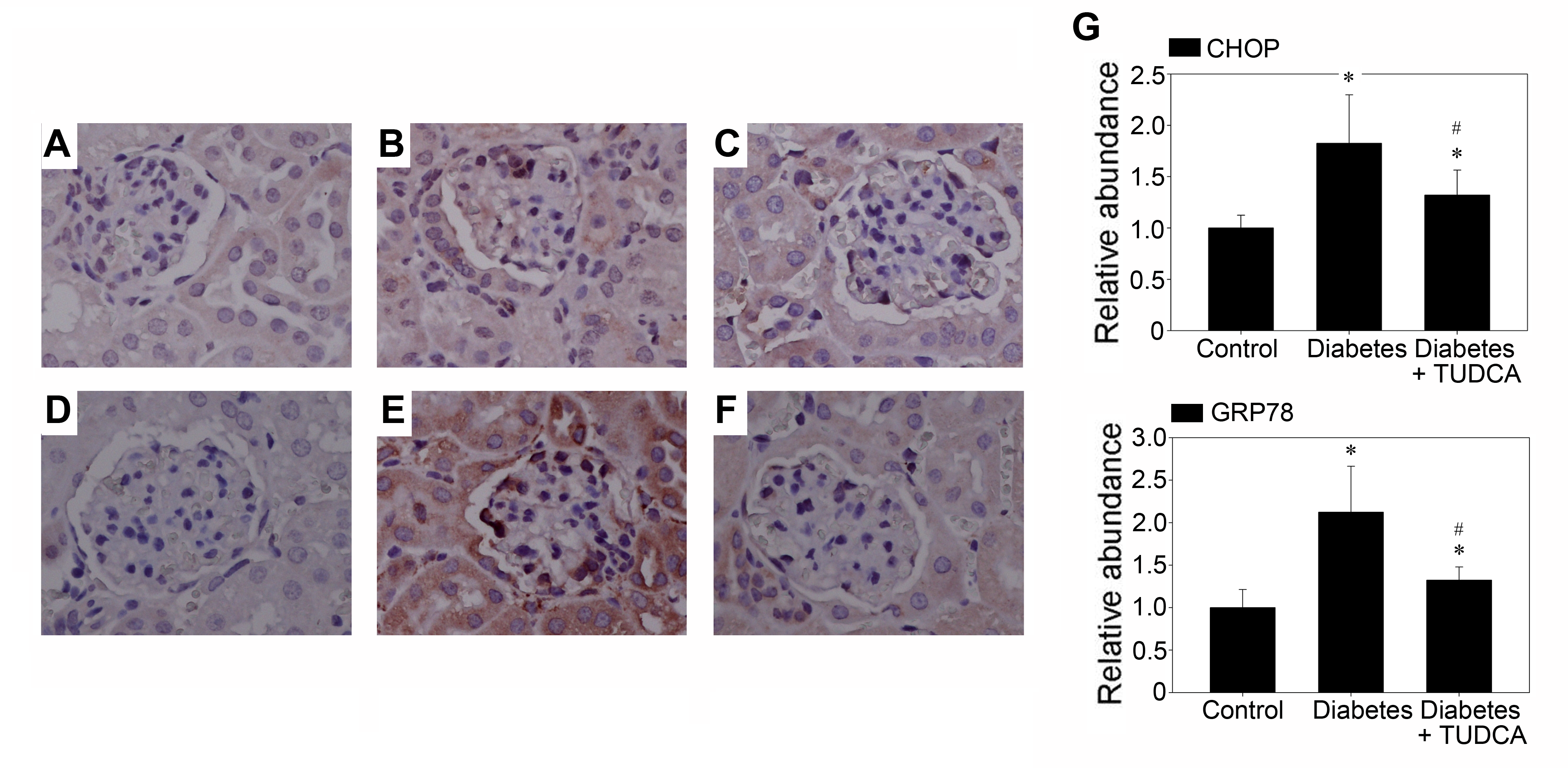

Supplement: Figure S2 — TUDCA attenuates ER stress in diabetic glomerular podocytes. (A–C) Representative micrographs by immunohistochemical staining of CHOP. A: control group; B: diabetic group; C: diabetic group treated with 500 mg/kg/day TUDCA. (D–F) Representative micrographs by immunohistochemical staining of GRP78. D: control group; E: diabetic group; F: diabetic group treated with 500 mg/kg/day TUDCA. G: The relative abundance of the semiquantitative histomorphometric analysis of the immunostaining of CHOP and GRP78. 30 glomeruli were evaluated for each experimental animal (n = 6). Data are presented as mean ± SEM. * P<0.05 vs. normal control. # P<0.05 vs. the group of 28 day diabetic mouse. (TIF) [file pone.0060546.s002.tif]
