# Supplementary material for: Autophagy Attenuates Diabetic Glomerular Damage through Protection of Hyperglycemia-Induced Podocyte Injury
Source: PLoS One. 2013 Apr 11;8(4):e60546. doi: 10.1371/journal.pone.0060546 (PMC3623813; doi:10.1371/journal.pone.0060546)
Supplement: Table S1 — Characteristics of the diabetic mice treated without or with TUDCA. (DOC) [file pone.0060546.s003.doc]

## Table S1. Characteristics of the diabetic mice treated without or with TUDCA.

|  | Control | Diabetic mouse | TUDCA therapy | |
| --- | --- | --- | --- | --- |
|  |  |  | 250mg/kg/day | 500mg/kg/day |
| Fast blood glucose (mmol/L) | 5.4±0.84 | 29.1±2.94*** | 28.3±2.59*** | 28.4±4.17*** |
| Urine albumin (mg/24h) | 30.2±5.41 | 143.3±13.62*** | 123.6±3.77***# | 102.4±6.57***# |
| Urine albumin/ Urine creatinine (mg/mg) | 1.68±0.642 | 12.32±2.829*** | 7.80±1.107***# | 3.80±1.226***# |
| Body weight (g) | 42.4±3.60 | 33.1±4.84*** | 35.0±3.71*** | 33.8±1.47*** |
| Kidney weight (g) | 0.36±0.056 | 0.39±0.072*** | 0.35±0.076# | 0.34±0.056# |
| Kidney / Body weight ratio | 0.008±0.0013 | 0.012±0.0022*** | 0.010±0.0021***# | 0.010±0.0015***# |

Values are expressed as the mean ± SEM. For each group, n=6. **P* < 0.05 vs. Control. # *P* < 0.05 vs. Diabetes.
